# Calcium Intake and Risk of Colorectal Cancer in the NIH-AARP Diet and Health Study

**DOI:** 10.1001/jamanetworkopen.2024.60283

**Published:** 2025-02-17

**Authors:** Semi Zouiouich, David Wahl, Linda M. Liao, Hyokyoung G. Hong, Rashmi Sinha, Erikka Loftfield

**Affiliations:** 1 Division of Cancer Epidemiology and Genetics, National Cancer Institute, Bethesda, Maryland

## Abstract

**Importance:**

Calcium intake is associated with a reduced risk of colorectal cancer (CRC), although it remains unclear whether this association varies by calcium source or tumor site. Moreover, there are disparities in calcium intake by race and ethnicity, but the impact of low calcium consumption on CRC risk in specific racial and ethnic populations is unclear.

**Objective:**

To investigate the association between calcium intake and CRC risk, considering the source of calcium and tumor site and across racial and ethnic groups.

**Design, Setting, and Participants:**

This cohort study analyzed data from the National Institutes of Health–AARP Diet and Health Study. Participants were aged 50 to 71 years at baseline (October 1995 to May 1996), had self-reported good health and neither extremely high nor low caloric or calcium intake, and were followed up until the date of their first primary cancer diagnosis, death, loss to follow-up, or end of follow-up (December 31, 2018). Data were analyzed from April 2022 to April 2024.

**Exposure:**

Calcium intake was estimated from dietary sources (dairy and nondairy), supplements, and total intake.

**Main Outcomes and Measures:**

The primary outcome was CRC incidence. Multivariable-adjusted Cox proportional hazards regression models estimated hazard ratios (HRs) and 95% CIs using the lowest sex-specific quintile of calcium intake as the reference.

**Results:**

Among 471 396 participants who were cancer-free at baseline, mean (SD) baseline age was 62.0 (5.4) years and 59.5% were male. During 7 339 055 person-years of follow-up (median, 18.4 years [IQR, 9.2-22.5 years]), 10 618 first primary CRC cases were identified. Mean (SD) total calcium intake for the lowest quintile (Q1) was 401 mg/d (104 mg/d) for females and 407 mg/d (95 mg/d) for males and for the highest quintile (Q5) was 2056 mg/d (412 mg/d) for females and 1773 mg/d (444 mg/d) for males. Dairy, nondairy, and supplemental sources contributed a mean (SD) of 42.1% (43.5%), 34.2% (24.5%), and 23.7% (38.3%) of total calcium intake, respectively. Higher total calcium intake (Q5 vs Q1) was associated with a lower risk of CRC (hazard ratio [HR], 0.71; 95% CI, 0.65-0.78; *P* < .001 for trend), with consistent results across calcium sources and tumor sites. Among non-Hispanic Black participants, the mean (SD) calcium intake was 382 mg/d (108 mg/d) for Q1 and 1916 mg/d (466 mg/d) for Q5, with no association of total calcium intake with CRC risk (Q5 vs Q1: HR, 0.60; 95% CI, 0.32-1.13; *P* = .12 for trend); there was no evidence of effect measure differences by race and ethnicity.

**Conclusions and Relevance:**

In this cohort study, higher calcium intake was consistently associated with reduced CRC risk across tumor sites and sources of calcium. Increasing calcium intake, especially among groups with lower consumption, may be associated with reductions in avoidable differences in CRC risk.

## Key Points

**Question:**

Is there an association between calcium intake and colorectal cancer risk, considering the source of calcium and tumor site?

**Findings:**

In this cohort study of 471 396 healthy adults with baseline age 50 to 71 years and more than 20 years of follow-up, an association between higher calcium intake and lower colorectal cancer risk was observed overall and by tumor site regardless of source of calcium.

**Meaning:**

Increasing calcium intake, particularly among population subgroups with lower intakes, may be associated with reductions in avoidable differences in colorectal cancer risk.

## Teaser

This cohort study uses data from the National Institutes of Health (NIH)–AARP Diet and Health Study to investigate whether intake of calcium from dietary sources and supplements is associated with risk of colorectal cancer (CRC).

## Introduction

Colorectal cancer (CRC) is the fourth most common cancer in the US, with an estimated 152 810 incident CRC cases and 53 010 CRC deaths in 2024.^1^ The World Cancer Research Fund Diet and Cancer Report 2018 concluded that evidence supports consumption of dairy products and calcium supplements for CRC prevention.^2^ In the US, close to 30% of men and 60% of women consume less than 1000 mg of calcium per day, making it a dietary component of public health concern for the general US population.^3^ Calcium deficiency can lead to osteoporosis and other bone disorders.^4^ Additionally, a nationally representative study found that levels of calcium insufficiency were higher among non-Hispanic Asian (48%) and non-Hispanic Black (47%) adults than among Hispanic (30%) and non-Hispanic White (24%) adults in the US.^5^

In the National Institutes of Health (NIH)–AARP Diet and Health Study, calcium intake has been associated with lower incident CRC risk.^6,7^ After an average of 7 years of follow-up, Park et al^6^ found that dairy food and calcium intake were inversely associated with CRC; however, associations were not reported by tumor site or by source of dietary calcium. Another analysis of a subset of NIH-AARP participants who provided data on adolescent and midlife diet found that higher calcium or milk intake in midlife regardless of intake in adolescence was associated with lower risk of colon cancer but not rectal cancer.^7^ With fewer than 3000 colon and 1000 rectal cancers, statistical power was limited,^7^ but previous research indicates that CRC tumor sites have distinct clinical and molecular characteristics as well as differential etiologies and risk factors.^8,9,10^

Now, with longer than 20 years of follow-up, more than 10 500 first primary CRC cases have been ascertained in the NIH-AARP cohort. We addressed outstanding questions to inform cancer prevention, including whether associations between calcium consumption and CRC differ by source of calcium intake, by tumor site, or across population subgroups, particularly those that have been documented as having high prevalence of calcium insufficiency.

## Methods

### Study Population

This cohort study used data from the NIH-AARP Study,^11^ which mailed self-administered questionnaires to 3.5 million AARP members aged 50 to 71 years who resided in 1 of 6 US states (California, Florida, Louisiana, New Jersey, North Carolina, or Pennsylvania) or 2 metropolitan areas (Atlanta, Georgia, or Detroit, Michigan) from October 1995 to May 1996. The NIH-AARP Study was approved by the Special Studies Institutional Review Board of the National Cancer Institute; informed consent was implied from completion and return of study questionnaires. The current study was exempted from review and informed consent per the Common Rule because all data used in the analysis were deidentified. All reported results adhered to the Strengthening the Reporting of Observational Studies in Epidemiology (STROBE) reporting guideline.

The baseline questionnaire queried about demographics, health-related behaviors, and dietary intake. Of the participants who satisfactorily returned the questionnaire, we excluded individuals who were proxy responders, had self-reported or prevalent cancer prior to baseline or had a death record only for cancer, had self-reported poor health or end-stage kidney disease, had no cancer registry report, reported extremely low or high caloric intake or calcium consumption (defined as >2 IQRs above the 75th percentile or below the 25th percentile of Box-Cox log-transformed intakes), or had no follow-up.

### Follow-Up and Cancer Outcome Ascertainment

Participants were followed up from baseline until the date of their first primary cancer diagnosis, death, loss to follow-up, or end of follow-up (December 31, 2018). CRC incidence was identified by probabilistic record linkage with the 8 state cancer registries that covered the original 6 states and 2 metropolitan areas plus 3 additional states (Arizona, Texas, and Nevada) to which participants moved during follow-up. CRC was defined by tumor and histologic code using the Surveillance, Epidemiology, and End Results Program incidence site recode and *International Classification of Diseases for Oncology, Third Edition* codes. We restricted our definition to primary adenocarcinoma of the proximal colon (cecum, C180; ascending colon, C182; hepatic flexure, C183; and transverse colon, C184), distal colon (splenic flexure, C185; descending colon, C186; and sigmoid colon, C187), and rectum (rectosigmoid junction, C199; rectum, C209) with the following histologic codes: 8140, 8141, 8143, 8145, 8210, 8211, 8221, 8260, 8261, 8262, 8263, 8470, 8480, 8481, and 8490. The following codes were censored in the site analysis: overlapping lesion of colon (C188), colon not otherwise specified (C189), and large intestine NOS (C260).

### Exposure Assessment

At baseline, study participants completed a self-administered questionnaire that included a 124-item food frequency questionnaire (FFQ).^11^ Participants reported their usual frequency of intake and portion size over the past 12 months. The food items, portion sizes, and nutrient database were constructed using the US Department of Agriculture 1994-1996 Continuing Survey of Food Intakes by Individuals.^12^ Participants reported frequency and types of multivitamins and frequency and dosage of individual calcium supplements, including antacids. Dietary calcium intake was estimated from diet only, including dairy (eg, milk, yogurt, cheese, cream, and ice cream) and nondairy calcium, and was adjusted for total energy intake using the nutrient density method (ie, milligrams per 1000 kilocalories per day). Supplemental calcium intake (milligrams per day) was estimated from multivitamins and calcium supplements and categorized into 4 groups: group 1, 0 mg/d; group 2, more than 0 to less than 400 mg/d; group 3, 400 to less than 1000 mg/d; or group 4, 1000 mg/d or more. Total calcium intake was calculated as the sum of dietary calcium (milligrams per day) and supplemental calcium (milligrams per day) intake.^13^ Dietary and total calcium intakes were categorized using sex-specific quintiles. Within a subset of participants who completed 2 nonconsecutive 24-hour dietary recalls within 1 year of baseline, energy-adjusted correlations between the FFQ and 24-hour recalls for estimated true calcium intake were 0.55 for men and 0.61 for women.^14^

### Statistical Analysis

We used Cox proportional hazards regression models to estimate hazard ratios (HRs) and 95% CIs for CRC by quintiles of calcium intake and continuously measured calcium intake for a fixed unit increase of 300 mg/d. The Cox proportional hazards regression models were adjusted for potential confounders. Models for total calcium were adjusted for sex; baseline age; race and ethnicity; educational level (high school or less, post–high school, some college, or college graduate or postgraduate); marital status (married, single); body mass index (calculated as weight in kilograms divided by height in meters squared; <25, ≥25 to <30, or ≥30); family history of cancer; smoking status and dose (never smoked, quit and smoked ≤20 cigarettes/d, quit and smoked >20 cigarettes/d, currently smoking ≤20 cigarettes/d, or currently smoking >20 cigarettes/d); physical activity (never or rarely, 1-3 times/mo, 1-2 times/wk, 3-4 times/wk, or ≥5 times per week); multivitamin use (never, 1-6 times/wk, 1 time/d, or >1 time/d); and intake of alcohol (0, <5, ≥5 to <15, ≥15 to <30, or ≥30 g/d), whole grains (servings per 1000 kcal/d), fruits and vegetables (cups per 1000 kcal/d), unprocessed red meat (g/1000 kcal/d), total processed meat (g/1000 kcal/d), supplemental folate (μg/1000 kcal/d), vitamin C (mg/1000 kcal/d), vitamin D (μg/1000 kcal/d), and total energy (kcal/d). Models for dietary calcium intake additionally adjusted for supplemental calcium intake, models for dairy calcium intake additionally adjusted for nondairy calcium intake, models for nondairy calcium intake additionally adjusted for dairy calcium intake, and models for supplemental calcium intake additionally adjusted for dairy and nondairy calcium intake. Dietary variables were nutrient density adjusted. Participant race and ethnicity were self-reported on the baseline questionnaire. Categories were American Indian or Alaska Native, Asian, or Pacific Islander; Hispanic; non-Hispanic Black (hereafter, Black); and non-Hispanic White (hereafter, White).

Missing values were included as indicator variables. No single variable had greater than 3.8% missing data. We used person-years as the underlying time metric. We conducted tests for linear trend across categories of calcium intake by assigning participants the midpoint of their calcium intake quintile and entering this single continuous variable into separate models. We tested the proportional hazards assumption by including an interaction term between calcium intake and person-years. We found no evidence that the assumption was violated for total calcium intake and CRC risk (*P* = .47).

In secondary analyses, we analyzed the association of CRC with calcium intake by source of calcium (ie, dairy and nondairy) overall and for each tumor site (ie, proximal colon, distal colon, and rectal cancer) and subsite (ie, cecum, ascending colon, hepatic flexure, transverse colon, splenic flexure, descending colon, sigmoid colon, rectosigmoid junction, and rectum). We stratified by racial and ethnic group to examine associations of different calcium sources with CRC risk. We performed a lag analysis for total calcium intake, considering CRC cases that occurred less than 5 years, 5 to 10 years, and more than 10 years after baseline.^15^

All analyses were performed from April 2022 to April 2024 using R, version 4.3.2 (R Core Team). Statistical tests were 2-sided, and *P* < .05 was interpreted as statistically significant.

## Results

Of 566 398 participants who satisfactorily returned the questionnaire, we excluded 15 760 proxy responders, 51 062 individuals with cancer prior to baseline or with a death record for cancer, 8365 with self-reported poor health, 769 with end-stage kidney disease, 14 113 with no cancer registry report, 3666 with extremely low or high reported caloric intake, 1220 with extremely low or high reported calcium consumption, and 47 with no follow-up. The analytic cohort consisted of 471 396 participants who were cancer-free at baseline. Among those, 10 618 incident first primary CRC cases were identified during 7 339 055 person-years of follow-up (median follow-up, 18.4 years [IQR, 9.2-22.5 years]). Mean (SD) age at baseline was 62.0 (5.4) years; 40.5% of participants were female, and 59.5% were male. A total of 1.7% of participants identified as American Indian or Alaska Native, Asian, or Pacific Islander; 3.8% as Black; 1.9% as Hispanic; and 91.3% as White (Table 1 and eTable 1 in Supplement 1).

**Table 1.** Characteristics of Participants at Baseline

Characteristic	Participants (N = 471 396)^a^		
	Quintile 1 (n = 94 282)	Quintile 3 (n = 94 281)	Quintile 5 (n = 94 278)
Total calcium, mg/d			
Female	401 (104)	971 (89)	2056 (412)
Male	407 (95)	831 (62)	1773 (444)
Dietary calcium, mg/d			
Female	363 (107)	694 (235)	1105 (560)
Male	381 (99)	733 (133)	1408 (527)
Dairy calcium, mg/d			
Female	141 (85)	370 (204)	711 (504)
Male	141 (79)	365 (144)	936 (515)
Nondairy calcium, mg/d			
Female	221 (77)	324 (130)	394 (193)
Male	240 (77)	368 (120)	472 (204)
Supplemental calcium, mg/d			
Female	38 (68)	277 (237)	950 (501)
Male	26 (57)	98 (125)	364 (420)
Baseline age, y	62.0 (5.4)	62.0 (5.4)	62.1 (5.4)
Race and ethnicity, No. (%)^b^			
American Indian or Alaska Native, Asian, or Pacific Islander	2240 (2.4)	1376 (1.5)	1270 (1.3)
Hispanic	1959 (2.1)	1703 (1.8)	1810 (1.9)
Non-Hispanic Black	5504 (5.8)	3393 (3.6)	2327 (2.5)
Non-Hispanic White	82 729 (87.7)	86 736 (92.0)	87 813 (93.1)
College graduate or postgraduate, No. (%)	33 144 (35.2)	36 992 (39.2)	39 344 (41.7)
Current BMI ≥30, No. (%)	20 558 (21.8)	20 131 (21.4)	19 250 (20.4)
First-degree relative with cancer, No. (%)	44 709 (47.4)	46 183 (49.0)	46 122 (48.9)
Never smoker, No. (%)	31 660 (33.6)	33 617 (35.7)	34 936 (37.1)
Physically active 3-4 times/wk, No. (%)	21 698 (23.0)	26 043 (27.6)	27 706 (29.4)
History of diabetes, No. (%)	7810 (8.3)	8149 (8.6)	8290 (8.8)
Current HRT use, No./total No. (%) of women	17 586/38 211 (46.0)	20 336/38 211 (53.2)	23 351/38 209 (61.1)
Alcohol consumption, g/d	11.4 (28.8)	12.8 (32.4)	12.0 (31.6)
Whole grains, servings/1000 kcal/d	0.509 (0.437)	0.584 (0.419)	0.595 (0.414)
Red meat, not processed, g/1000 kcal/d	29.0 (19.1)	25.9 (16.9)	22.2 (15.8)
Processed meat, g/1000 kcal/d	11.4 (11.4)	10.6 (9.9)	9.12 (9.1)
Vitamin D, μg/1000 kcal/d	1.83 (0.96)	2.43 (1.20)	3.48 (1.88)
Total energy, kcal/d	1260 (467)	1850 (666)	2360 (962)

Abbreviations: BMI, body mass index (calculated as weight in kilograms divided by height in meters squared); HRT, hormone replacement therapy.

^a^ Data are presented as mean (SD) unless otherwise indicated.

^b^ Data were missing for 6136 participants (1.3%).

Mean (SD) total calcium intake was 401 mg/d (104 mg/d) for females and 407 mg/d (95 mg/d) for males in the lowest quintile (Q1) and 2056 mg/d (412 mg/d) for females and 1773 mg/d (444 mg/d) for males in the highest quintile (Q5). Total calcium intake ranged from 105 to 5010 mg/d; dairy intake, from 0 to 3316 mg/d; nondairy intake, from 19 to 2509 mg/d; and supplemental intake, from 0 to 1662 mg/d (eTable 2 in Supplement 1). Dairy, nondairy, and supplemental sources contributed a mean (SD) of 42.1% (43.5%), 34.2% (24.5%), and 23.7% (38.3%) of total calcium intake, respectively.

**Figure 1.**
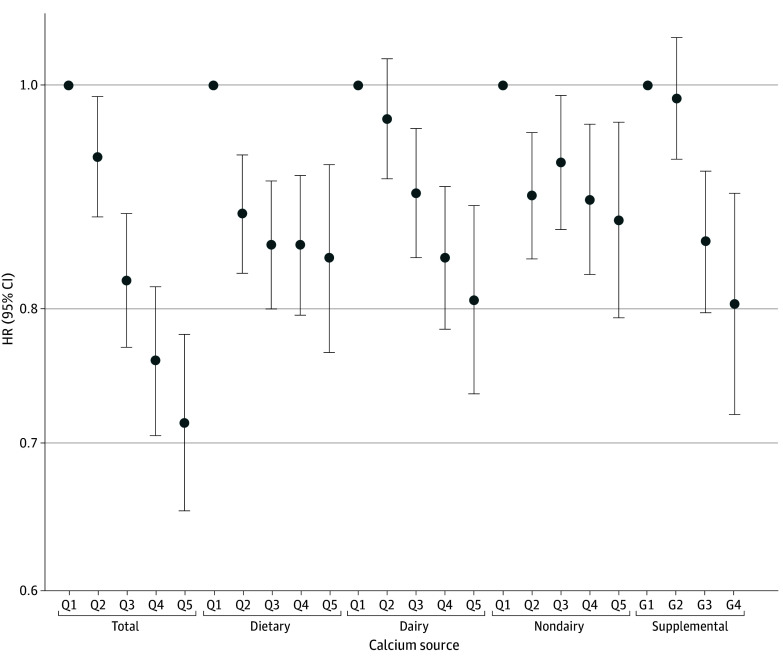
Associations of Sex-Specific Total, Dietary, Dairy, Nondairy, and Supplemental Calcium Intake With Colorectal Cancer Incidence Quintile 1 (Q1) was the lowest, and Q5 was the highest. Supplemental calcium intake was categorized into 4 groups: 0 mg/d (group 1 [G1]), more than 0 to less than 400 mg/d (G2), 400 to less than 1000 mg/d (G3), and 1000 mg/d or more (G4). Adjustment factors are given in the Statistical Analysis subsection of the Methods section. HR indicates hazard ratio.

Total calcium (Q5 vs Q1: HR, 0.71; 95% CI, 0.65-0.78; *P* < .001 for trend), dietary calcium (Q5 vs Q1: HR, 0.84; 95% CI, 0.77-0.92; *P* = .001 for trend), and supplemental calcium (≥1000 mg/d vs 0 to <400 mg/d: HR, 0.80; 95% CI, 0.72-0.90; *P* < .001 for trend) intake were associated with lower risk of CRC after adjustment for potential confounders (Figure 1 and eTable 3 in Supplement 1). The HR estimates for total calcium intake and CRC risk were similar in females and males (eTable 4 in Supplement 1). There was a reduced risk of CRC for every additional 300 mg/d of total (HR, 0.92; 95% CI, 0.90-0.95), dietary (HR, 0.90; 95% CI, 0.84-0.96), and supplemental (HR, 0.95; 95% CI, 0.93-0.97) calcium consumed (eTable 5 in Supplement 1).

**Figure 2.**
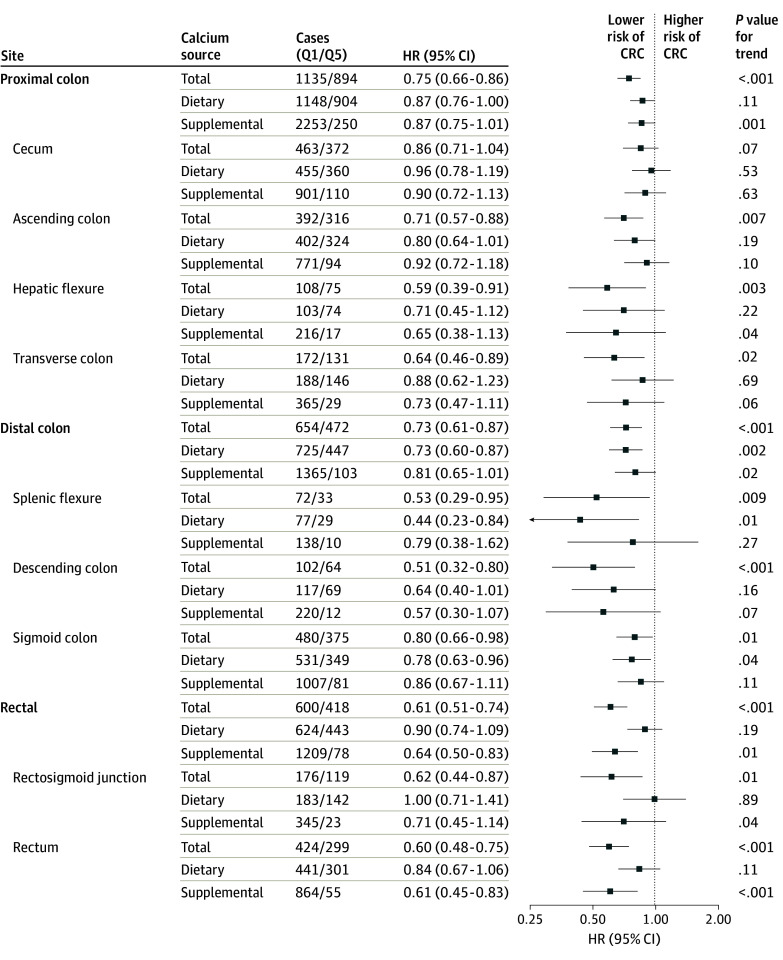
Associations of Total, Dietary, and Supplemental Calcium Intake Quintiles With Cancer Incidence by Anatomical Tumor Site Within the Colon and Rectum Quintile 1 (Q1) was the lowest, and Q5 was the highest. For supplemental calcium intake, cases are given as group 1 (0 mg/d)/group 4 (≥1000 mg/d). Adjustment factors are given in the Statistical Analysis subsection of the Methods section. CRC indicates colorectal cancer; HR, hazard ratio.

The HR estimates were consistent by tumor site; total calcium was associated with lower risk of proximal colon (Q5 vs Q1: HR, 0.75; 95% CI, 0.66-0.86; *P* < .001 for trend), distal colon (Q5 vs Q1: HR, 0.73; 95% CI, 0.61-0.87; *P* < .001 for trend), and rectal (Q5 vs Q1: HR, 0.61; 95% CI, 0.51-0.74; *P* < .001 for trend) cancer (Figure 2 and eTable 6 in Supplement 1). Except for tumors located in the cecum, we observed statistically significant associations between total calcium intake and cancer risk for each subsite (Figure 2 and eTable 6 in Supplement 1). Associations between dietary calcium and CRC risk for each tumor subsite were generally inverse, but case numbers were more limited (Figure 2 and eTable 7 in Supplement 1). There was no association between supplemental calcium intake and risk of proximal colon (group 4 vs 1: HR, 0.87; 95% CI, 0.75-1.01; *P* = .001 for trend) and distal colon (group 4 vs 1: HR, 0.81; 95% CI, 0.65-1.01; *P* = .02 for trend) cancer, but there was an association with lower risk of rectal cancer (group 4 vs 1: HR, 0.65; 95% CI, 0.50-0.83; *P* = .01) (Figure 2 and eTable 8 in Supplement 1).

**Figure 3.**
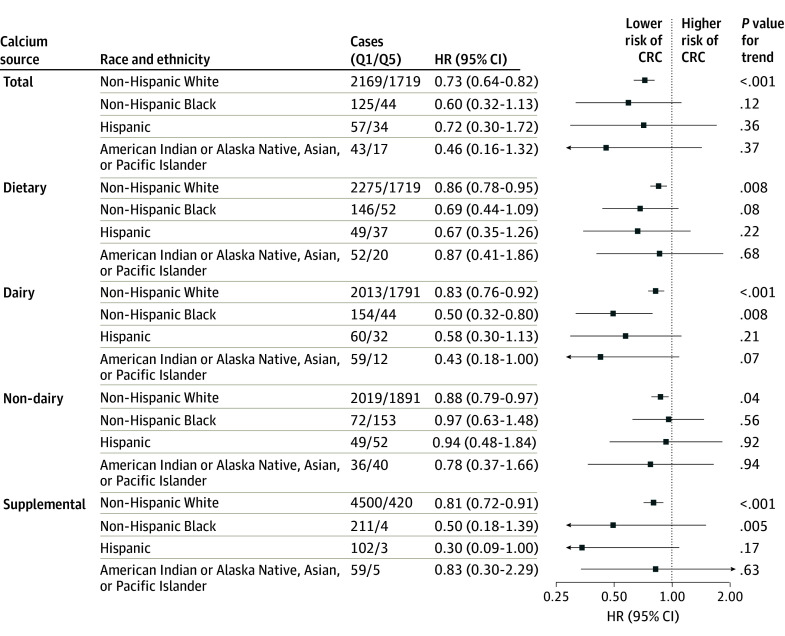
Associations of Total, Dietary, and Supplemental Calcium Intake With Colorectal Cancer Incidence by Race and Ethnicity Quintile 1 (Q1) was the lowest, and Q5 was the highest. For supplemental calcium intake, cases are given as group 1 (0 mg/d)/group 4 (≥1000 mg/d). Adjustment factors are given in the Statistical Analysis subsection of the Methods section. HR indicates hazard ratio.

The HR estimates were consistent by race and ethnicity, with no evidence of effect measure differences (*P* = .63 for heterogeneity). Total calcium (Q5 vs Q1: HR, 0.73; 95% CI, 0.64-0.82; *P* < .001 for trend), dietary calcium (Q5 vs Q1: HR, 0.86; 95% CI, 0.78-0.95; *P* = .008 for trend), and supplemental calcium (group 4 vs 1: HR, 0.81; 95% CI, 0.72-0.91; *P* < .001 for trend) intake were associated with lower CRC risk in White participants (Figure 3 and eTables 5 and 9 in Supplement 1). Among Black participants (n = 18 095; 401 CRC cases), mean (SD) total calcium intake was 382 mg/d (108 mg/d) in Q1 and 1916 mg/d (466 mg/d) in Q5, and dairy, nondairy, and supplemental sources accounted for a mean (SD) of 35.5% (43.5%), 45.4% (36.8%), and 19.1% (32.8%) of total calcium, respectively. Dairy calcium intake among Black participants was inversely associated with risk of CRC (Q5 vs Q1: HR, 0.50; 95% CI, 0.32-0.80; *P* = .008 for trend), but total calcium intake was not associated with CRC risk (Q5 vs Q1: HR, 0.60; 95% CI, 0.32-1.13; *P* = .12 for trend). Among Black individuals, the risk of CRC was reduced for every additional 300 mg/d of total (HR, 0.68; 95% CI, 0.56-0.82), dietary (HR, 0.64; 95% CI, 0.49-0.85), and supplemental (HR, 0.81; 95% CI, 0.69-0.96) calcium consumed (eTable 5 in Supplement 1).

To explore whether HR estimates varied over time, we evaluated calcium-CRC associations during different follow-up periods (Table 2). Higher calcium intake was consistently associated with lower CRC risk over the course of follow-up.

**Table 2.** AHRs and 95% CIs for CRC Incidence by Quintiles of Total Calcium Intake and Lag Time^a^

Lag time	Total calcium intake quintile					*P* value for trend
	1	2	3	4	5	
**Overall (N = 471 396)**						
AHR (95% CI)	1 [Reference]	0.93 (0.88-0.99)	0.82 (0.77-0.88)	0.76 (0.71-0.82)	0.71 (0.65-0.78)	<.001
Cases, No.	2444	2320	2077	1940	1837	NA
**<5 y of Follow-up (n = 471 396)**						
AHR (95% CI)	1 [Reference]	0.96 (0.84-1.08)	0.80 (0.69-0.92)	0.81 (0.69-0.94)	0.75 (0.63-0.91)	.001
Cases, No.	556	535	454	461	440	NA
**5-10 y of Follow-up (n = 423 195)**						
AHR (95% CI)	1 [Reference]	0.86 (0.77-0.97)	0.76 (0.67-0.86)	0.68 (0.59-0.78)	0.62 (0.52-0.73)	<.001
Cases, No.	739	652	582	530	486	NA
**>10 y of Follow-up (n = 355 747)**						
AHR (95% CI)	1 [Reference]	0.96 (0.88-1.05)	0.87 (0.80-0.96)	0.79 (0.71-0.88)	0.76 (0.67-0.86)	<.001
Cases, No.	1149	1133	1041	949	911	NA

Abbreviations: AHR, adjusted hazard ratio; CRC, colorectal cancer; NA, not applicable.

^a^ Adjusted for sex; baseline age; race and ethnicity; educational level; marital status; body mass index; family history of cancer; smoking dose and status; physical activity; multivitamin use; and intake of alcohol, whole grains, fruits and vegetables, unprocessed red meat, total processed meat, supplemental folate, vitamin C, vitamin D, and total energy.

## Discussion

To our knowledge, this cohort study investigating calcium source and tumor site was the most extensive analysis to date on the association of calcium intake with incident CRC. With 10 618 first primary CRC cases diagnosed over 23 years of follow-up, we explored calcium-CRC associations across population subgroups that have been identified as having lower calcium intake in the general US population, namely women and racial and ethnic groups.^5^ We found a dose-response relationship between higher calcium intake and lower relative risk of incident CRC overall, by tumor site, and across population subgroups regardless of source of calcium. Overall, for each additional 300 mg/d in total, dietary, and supplemental calcium intake, there was an 8%, 10%, and 5% decrease in CRC risk, respectively, and in Black individuals, there was a 32%, 36%, and 19% decrease in CRC risk, respectively.

Our results are consistent with previous studies showing that higher calcium intake is associated with lower CRC risk.^16,17,18^ In a pooled analysis of 10 cohort studies, when comparing individuals in the highest and the lowest quintile of calcium intake, total and dietary calcium intakes were associated with a 22% and 14% lower CRC risk, respectively.^19^ Our results are also consistent with a review that found that for every additional 300 mg/d of total and supplemental calcium consumed, there was an 8% and 9% lower CRC risk, respectively.^20^ We extended these prior findings by demonstrating that higher dairy and nondairy calcium intakes were also associated with lower CRC risk.

Prior studies have generally focused on calcium intake from dairy sources.^19,21,22^ One dairy-specific hypothesis is that calcium in dairy products binds to secondary bile acids and fatty acids in the colon, reducing their potential to promote cancer development.^23^ Additionally, dairy products contain vitamin D, which may increase absorption of calcium in the gastrointestinal tract.^24^ However, the relationship between nondairy dietary calcium intake and CRC is less clear. Contrary to our findings, some studies found no evidence of an inverse association between nondairy dietary calcium and CRC.^24,25,26^ One potential explanation is that plant-based calcium sources often contain oxalate, cellulose, and phytate, compounds known to hinder calcium absorption.^27,28^ Nondairy sources generally contribute less to dietary calcium intake than dairy sources. Thus, prior studies of calcium intake from nondairy sources may have lacked an adequate intake range to observe an association. A systematic review and meta-analysis of randomized clinical trials investigating supplemental calcium and colorectal adenomas generally supported an association of intake with decreased risk over a follow-up period of 3 to 5 years.^29^ However, 1 randomized chemoprevention trial in patients with colorectal adenomas found that calcium and vitamin D supplementation increased sessile serrated adenoma or polyp risk 6 to 10 years after supplementation.^30^ Participants in 1 treatment group were given 1200 mg/d of supplemental calcium in addition to their dietary calcium intake (mean, 718 mg/d), resulting in a higher intake of total calcium than in our study. Still, in the NIH-AARP study, those in the highest category of total calcium intake had lower CRC risk earlier and later during follow-up, and the inverse association did not differ by sex or anatomic location of CRC; this would have been expected if calcium intake had increased risk of sessile serrated adenomas or polyps, which are more common in females and in the proximal colon.^31,32^

Epidemiologic research on calcium intake and site-specific colon cancer risk has been inconclusive. A meta-analysis reported greater risk reduction associated with tumors in the distal colon and rectum than with tumors in the proximal colon.^21^ However, these findings may have been due to limited case numbers in individual studies rather than varying etiologies. Proximal and distal colonic sites differ in embryologic origin, physiologic function, fecal composition, and transit times.^33^ In addition, some risk factors for CRC are site specific, such as physical activity, anthropometry, and smoking.^26^ Variability at the molecular level has also been observed in CRC tumors across anatomic sites. For example, the WNT signaling pathway plays a crucial role in regulating cell proliferation and differentiation.^34^ The distal colon has higher WNT signaling activity, potentially making it more responsive to factors that modulate this pathway, such as calcium intake.^35,36^ Thus, enhanced cell differentiation in response to calcium could help maintain tissue integrity and reduce the risk of cancerous cell growth. While our study found an inverse association of total and supplemental calcium intake with rectal cancer risk, we did not observe a statistically significant association with dietary calcium intake. This discrepancy may have resulted from unique environmental or biological differences in the rectum compared with other colorectal subsites. For instance, prolonged exposure to fecal matter in the rectum and a distinct microbial composition could influence local availability and bioactivity of calcium.^10,37^ Additionally, the aforementioned variations in calcium bioavailability from dietary sources could further modulate its impact specifically in the rectum.^27,28^ Nevertheless, with a substantial number of first primary CRC cases, we found a dose-response relationship between higher calcium intake and lower CRC risk regardless of tumor site, suggesting that the mechanisms underlying the association between calcium intake and CRC risk may not be site specific.

Calcium insufficiency is higher in certain US population subgroups, including female, non-Hispanic Black, and non-Hispanic Asian populations.^5^ According to previous studies, non-Hispanic Black individuals tend to have lower calcium consumption from dietary sources owing to higher incidence of lactose intolerance and limited use of supplements.^38,39,40^ In our analysis, we observed no evidence of effect measure differences by racial and ethnic group. However, in line with national US data,^5^ we found that Black participants in our study had a lower range of total calcium intake than White participants.

### Limitations

Our study has limitations. Dietary information was collected via the FFQ for the full cohort at baseline only; thus, we were unable to assess changes in calcium intake over time. Although measurement error is inherent to FFQ-based dietary measures, FFQ-derived calcium intake was highly correlated with estimated true intake, and misclassification of our exposure was likely to be nondifferential owing to our prospective study design.^41^ Limited statistical power owing to smaller case numbers for anatomic subsites precluded subsite analyses stratified by sex or race and ethnicity. Still, to our knowledge, this was the largest cohort study of calcium intake and CRC risk and one of the only studies to explore variation by calcium source and tumor subsite. The maximum 23-year follow-up period allowed us to explore associations between calcium and CRC incidence during different follow-up periods, and we found inverse associations between total calcium intake and CRC risk irrespective of whether the cases occurred within 5 years of baseline or more than a decade later, suggesting that reverse causality is an unlikely explanation for the observed associations.

## Conclusions

In this cohort study, higher calcium intake was associated with lower CRC risk overall regardless of source of calcium or tumor site. While calcium intake may vary by race and ethnicity, the potential for calcium to play a role in CRC prevention appeared to be consistent across racial and ethnic groups; still, research in racial and ethnic minority populations is needed. Increasing calcium intake, particularly among population subgroups with lower intakes, may be associated with a reduction in avoidable differences in CRC cancer risk.

## Notes

Supplement 1. **eTable 1.** Characteristics of Participants at Baseline **eTable 2.** Distribution of Calcium Intake by Sex-Specific Quintiles in Each Calcium Source for Males and Females **eTable 3.** Adjusted Associations of Colorectal Cancer Incidence With Sex-Specific Quintiles of Total, Dietary, Dairy, and Nondairy Calcium and Categories of Supplemental Calcium in the NIH-AARP Diet and Health Study **eTable 4.** Adjusted Associations of Colorectal Cancer Incidence With Sex-Specific Quintiles of Total, Dietary, Dairy, and Nondairy Calcium and Categories of Supplemental Calcium by Sex in the NIH-AARP Diet and Health Study **eTable 5.** Adjusted Associations of Colorectal Cancer Incidence With Sex-Specific Quintiles of Total, Dietary, Dairy, and Nondairy Calcium and Categories of Supplemental Calcium by Race and Ethnicity in the NIH-AARP Diet and Health Study **eTable 6.** Adjusted Associations of Colorectal Cancer Incidence With Quintiles of Total Calcium by Tumor Site of Colon and Rectum in the NIH-AARP Diet and Health Study **eTable 7.** Adjusted Associations of Colorectal Cancer Incidence With Quintiles of Dietary Calcium by Tumor Site of Colon and Rectum in the NIH-AARP Diet and Health Study **eTable 8.** Adjusted Associations of Colorectal Cancer Incidence With Categories of Supplemental Calcium by Tumor Site of Colon and Rectum in the NIH-AARP Diet and Health Study **eTable 9.** Adjusted Associations of Colorectal Cancer Incidence With Quintiles of Total and Dietary Calcium and Categories of Supplemental Calcium in Participants Who Were American Indian or Alaska Native, Asian, or Pacific Islander; Hispanic; Non-Hispanic Black; or Non-Hispanic White in the NIH-AARP Diet and Health Study

Supplement 2.
Data Sharing Statement
