# Supplementary material for: Calcium Intake and Risk of Colorectal Cancer in the NIH-AARP Diet and Health Study
Source: JAMA Netw Open. 2025 Feb 17;8(2):e2460283. doi: 10.1001/jamanetworkopen.2024.60283 (PMC11833519; doi:10.1001/jamanetworkopen.2024.60283)
Supplement: Supplement 1. — eTable 1. Characteristics of Participants at Baseline eTable 2. Distribution of Calcium Intake by Sex-Specific Quintiles in Each Calcium Source for Males and Females eTable 3. Adjusted Associations of Colorectal Cancer Incidence With Sex-Specific Quintiles of Total, Dietary, Dairy, and Nondairy Calcium and Categories of Supplemental Calcium in the NIH-AARP Diet and Health Study eTable 4. Adjusted Associations of Colorectal Cancer Incidence With Sex-Specific Quintiles of Total, Dietary, Dairy, and Nondairy Calcium and Categories of Supplemental Calcium by Sex in the NIH-AARP Diet and Health Study eTable 5. Adjusted Associations of Colorectal Cancer Incidence With Sex-Specific Quintiles of Total, Dietary, Dairy, and Nondairy Calcium and Categories of Supplemental Calcium by Race and Ethnicity in the NIH-AARP Diet and Health Study eTable 6. Adjusted Associations of Colorectal Cancer Incidence With Quintiles of Total Calcium by Tumor Site of Colon and Rectum in the NIH-AARP Diet and Health Study eTable 7. Adjusted Associations of Colorectal Cancer Incidence With Quintiles of Dietary Calcium by Tumor Site of Colon and Rectum in the NIH-AARP Diet and Health Study eTable 8. Adjusted Associations of Colorectal Cancer Incidence With Categories of Supplemental Calcium by Tumor Site of Colon and Rectum in the NIH-AARP Diet and Health Study eTable 9. Adjusted Associations of Colorectal Cancer Incidence With Quintiles of Total and Dietary Calcium and Categories of Supplemental Calcium in Participants Who Were American Indian or Alaska Native, Asian, or Pacific Islander; Hispanic; Non-Hispanic Black; or Non-Hispanic White in the NIH-AARP Diet and Health Study [file jamanetwopen-e2460283-s001.pdf]

Supplemental Online Content

Zouiouich S, Wahl D, Liao LM, Hong HG, Sinha R, Loftfield E. Calcium intake and risk of colorectal cancer in the NIH-AARP Diet and Health Study. *JAMA Netw Open.* 2025;8(2):e2460283. doi:10.1001/jamanetworkopen.2024.60283

eTable 1. Characteristics of Participants at Baseline

eTable 2. Distribution of Calcium Intake by Sex-Specific Quintiles in Each Calcium Source for Males and Females

eTable 3. Adjusted Associations of Colorectal Cancer Incidence With Sex-Specific Quintiles of Total, Dietary, Dairy, and Nondairy Calcium and Categories of Supplemental Calcium in the NIH-AARP Diet and Health Study

eTable 4. Adjusted Associations of Colorectal Cancer Incidence With Sex-Specific Quintiles of Total, Dietary, Dairy, and Nondairy Calcium and Categories of Supplemental Calcium by Sex in the NIH-AARP Diet and Health Study

eTable 5. Adjusted Associations of Colorectal Cancer Incidence With Continuous Total, Dietary, Dairy, Nondairy, and Supplemental Calcium by Race and Ethnicity in the NIH-AARP Diet and Health Study

eTable 6. Adjusted Associations of Colorectal Cancer Incidence With Quintiles of Total Calcium by Tumor Site of Colon and Rectum in the NIH-AARP Diet and Health Study

eTable 7. Adjusted Associations of Colorectal Cancer Incidence With Quintiles of Dietary Calcium by Tumor Site of Colon and Rectum in the NIH-AARP Diet and Health Study

eTable 8. Adjusted Associations of Colorectal Cancer Incidence With Categories of Supplemental Calcium by Tumor Site of Colon and Rectum in the NIH-AARP Diet and Health Study

eTable 9. Adjusted Associations of Colorectal Cancer Incidence With Quintiles of Total, Dietary, Dairy, and Nondairy Calcium and Categories of Supplemental Calcium in Participants Who Were American Indian or Alaska Native, Asian, or Pacific Islander; Hispanic; Non-Hispanic Black; or Non-Hispanic White in the NIH-AARP Diet and Health Study

© 2025 Zouiouich S et al. *JAMA Network Open*.

This supplemental material has been provided by the authors to give readers additional information about their work.

© 2025 Zouiouich S et al. *JAMA Network Open*.

eTable 1. Characteristics of Participants at Baseline

	**Total calcium (both sex)**					**Male**					**Female**				
	**Quintile 1**	**Quintile 2**	**Quintile 3**	**Quintile 4**	**Quintile 5**	**Quintile 1**	**Quintile 2**	**Quintile 3**	**Quintile 4**	**Quintile 5**	**Quintile 1**	**Quintile 2**	**Quintile 3**	**Quintile 4**	**Quintile 5**
	**(N=94282)**	**(N=94277)**	**(N=94281)**	**(N=94278)**	**(N=94278)**	**(N=56071)**	**(N=56069)**	**(N=56070)**	**(N=56069)**	**(N=56069)**	**(N=38211)**	**(N=38208)**	**(N=38211)**	**(N=38209)**	**(N=38209)**
**Total calcium (mg/day)**															
Mean (SD)	404 (98.8)	655 (69.2)	888 (101)	1197 (165)	1887 (453)	407 (94.8)	633 (54.5)	831 (62.0)	1097 (97.5)	1773 (444)	401 (104)	686 (76.2)	971 (89.3)	1344 (131)	2056 (412)
Median [Min, Max]	422 [105, 555]	651 [538, 821]	875 [728, 1132]	1180 [944, 1586]	1792 [1287, 5010]	425 [105, 538]	634 [538, 728]	829 [728, 944]	1088 [944, 1287]	1650 [1287, 4939]	416 [106, 555]	685 [555, 821]	968 [821, 1132]	1337 [1132, 1586]	1952 [1586, 5010]
**Dietary Calcium (mg/day)**															
Mean (SD)	373 (102)	571 (118)	717 (182)	890 (273)	1286 (561)	381 (98.7)	572 (96.8)	733 (133)	928 (211)	1408 (527)	363 (107)	569 (143)	694 (235)	835 (337)	1105 (560)
Median [Min, Max]	377 [105, 555]	582 [106, 821]	748 [107, 1132]	939 [106, 1586]	1272 [107, 3468]	386 [105, 538]	579 [108, 728]	753 [112, 944]	970 [110, 1287]	1373 [118, 3468]	365 [106, 555]	587 [106, 821]	720 [107, 1132]	807 [106, 1586]	1002 [107, 3466]
**Dairy calcium (mg/day)**															

© 2025 Zouiouich S et al. *JAMA Network Open*.

Mean (SD)	141 (81.8)	263 (118)	367 (171)	502 (250)	845 (522)	141 (79.3)	254 (106)	365 (144)	513 (211)	936 (515)	141 (85.4)	275 (132)	370 (204)	487 (299)	711 (504)
Median [Min, Max]	131 [0, 498]	261 [0, 719]	364 [0, 1074]	498 [0, 1447]	780 [0, 3316]	132 [0, 445]	253 [0, 658]	366 [0, 828]	525 [0, 1224]	875 [0, 3316]	129 [0, 498]	274 [0, 719]	357 [0, 1074]	441 [0, 1447]	594 [0, 3270]
**Non-dairy calcium (mg/day)**															
Mean (SD)	232 (77.5)	308 (99.0)	350 (126)	388 (155)	440 (203)	240 (76.8)	318 (94.0)	368 (120)	415 (149)	472 (204)	221 (77.2)	293 (104)	324 (130)	348 (154)	394 (193)
Median [Min, Max]	226 [22.5, 553]	299 [18.6, 820]	336 [28.3, 1086]	364 [28.8, 1414]	400 [31.5, 2509]	235 [22.5, 536]	310 [18.6, 716]	356 [29.2, 941]	393 [40.7, 1269]	435 [31.5, 2509]	214 [26.4, 553]	282 [30.2, 820]	303 [28.3, 1086]	319 [28.8, 1414]	352 [36.1, 2171]
**Supplemental calcium (mg/day)**															
Mean (SD)	31.2 (61.7)	84.0 (114)	170 (199)	307 (318)	602 (538)	26.3 (56.7)	61.2 (85.9)	97.7 (125)	169 (201)	364 (420)	38.3 (67.8)	117 (140)	277 (237)	509 (350)	950 (501)
Median [Min, Max]	0 [0, 412]	0 [0, 662]	162 [0, 1000]	162 [0, 1234]	500 [0, 1662]	0 [0, 412]	0 [0, 546]	46.3 [0, 715]	162 [0, 1162]	162 [0, 1662]	0 [0, 412]	71.5 [0, 662]	187 [0, 1000]	500 [0, 1234]	1000 [0, 1662]
**Baseline age (years)**															
Mean (SD)	62.0 (5.39)	61.9 (5.38)	62.0 (5.37)	62.1 (5.36)	62.1 (5.36)	62.1 (5.37)	62.1 (5.35)	62.1 (5.35)	62.1 (5.35)	62.2 (5.35)	61.8 (5.41)	61.7 (5.41)	61.8 (5.40)	61.9 (5.38)	62.0 (5.39)

© 2025 Zouiouich S et al. *JAMA Network Open*.

Median [Min, Max]	62.5 [50.3, 71.5]	62.5 [50.3, 71.4]	62.6 [50.3, 71.5]	62.6 [50.3, 71.5]	62.7 [50.3, 71.5]	62.7 [50.3, 71.5]	62.7 [50.3, 71.4]	62.8 [50.3, 71.5]	62.7 [50.3, 71.5]	62.8 [50.4, 71.5]	62.2 [50.3, 71.5]	62.1 [50.3, 71.4]	62.3 [50.4, 71.5]	62.4 [50.4, 71.5]	62.5 [50.3, 71.3]
**Race (%)**															
Non-Hispanic White	82729 (87.7%)	85945 (91.2%)	86736 (92.0%)	87118 (92.4%)	87813 (93.1%)	50016 (89.2%)	52128 (93.0%)	52452 (93.5%)	52388 (93.4%)	52516 (93.7%)	32713 (85.6%)	33817 (88.5%)	34284 (89.7%)	34730 (90.9%)	35297 (92.4%)
Non-Hispanic Black	5504 (5.8%)	3931 (4.2%)	3393 (3.6%)	2940 (3.1%)	2327 (2.5%)	2318 (4.1%)	1432 (2.6%)	1328 (2.4%)	1262 (2.3%)	1102 (2.0%)	3186 (8.3%)	2499 (6.5%)	2065 (5.4%)	1678 (4.4%)	1225 (3.2%)
Hispanic	1959 (2.1%)	1724 (1.8%)	1703 (1.8%)	1817 (1.9%)	1810 (1.9%)	1198 (2.1%)	993 (1.8%)	970 (1.7%)	1070 (1.9%)	1120 (2.0%)	761 (2.0%)	731 (1.9%)	733 (1.9%)	747 (2.0%)	690 (1.8%)
Asian, Pacific Islander, Native American	2240 (2.4%)	1550 (1.6%)	1376 (1.5%)	1375 (1.5%)	1270 (1.3%)	1535 (2.7%)	958 (1.7%)	780 (1.4%)	796 (1.4%)	733 (1.3%)	705 (1.8%)	592 (1.5%)	596 (1.6%)	579 (1.5%)	537 (1.4%)
Missing	1850 (2.0%)	1127 (1.2%)	1073 (1.1%)	1028 (1.1%)	1058 (1.1%)	1004 (1.8%)	558 (1.0%)	540 (1.0%)	553 (1.0%)	598 (1.1%)	846 (2.2%)	569 (1.5%)	533 (1.4%)	475 (1.2%)	460 (1.2%)
**Education (%)**															
High school or less	26471 (28.1%)	24756 (26.3%)	23576 (25.0%)	22955 (24.3%)	21261 (22.6%)	12655 (22.6%)	11891 (21.2%)	11598 (20.7%)	11598 (20.7%)	11483 (20.5%)	13816 (36.2%)	12865 (33.7%)	11978 (31.3%)	11357 (29.7%)	9778 (25.6%)
Post high school	9192 (9.7%)	9478 (10.1%)	9416 (10.0%)	9188 (9.7%)	9001 (9.5%)	5116 (9.1%)	5278 (9.4%)	5257 (9.4%)	5238 (9.3%)	5057 (9.0%)	4076 (10.7%)	4200 (11.0%)	4159 (10.9%)	3950 (10.3%)	3944 (10.3%)

© 2025 Zouiouich S et al. *JAMA Network Open*.

Some college	22345 (23.7%)	21725 (23.0%)	21765 (23.1%)	21401 (22.7%)	21877 (23.2%)	12912 (23.0%)	12393 (22.1%)	12334 (22.0%)	12024 (21.4%)	12222 (21.8%)	9433 (24.7%)	9332 (24.4%)	9431 (24.7%)	9377 (24.5%)	9655 (25.3%)
College and post graduate	33144 (35.2%)	35822 (38.0%)	36992 (39.2%)	38150 (40.5%)	39344 (41.7%)	23703 (42.3%)	25217 (45.0%)	25532 (45.5%)	25791 (46.0%)	25745 (45.9%)	9441 (24.7%)	10605 (27.8%)	11460 (30.0%)	12359 (32.3%)	13599 (35.6%)
Missing	3130 (3.3%)	2496 (2.6%)	2532 (2.7%)	2584 (2.7%)	2795 (3.0%)	1685 (3.0%)	1290 (2.3%)	1349 (2.4%)	1418 (2.5%)	1562 (2.8%)	1445 (3.8%)	1206 (3.2%)	1183 (3.1%)	1166 (3.1%)	1233 (3.2%)
**Marital status (%)**															
Single	29665 (31.5%)	28312 (30.0%)	28178 (29.9%)	28494 (30.2%)	29195 (31.0%)	8340 (14.9%)	7630 (13.6%)	7502 (13.4%)	7755 (13.8%)	8434 (15.0%)	21325 (55.8%)	20682 (54.1%)	20676 (54.1%)	20739 (54.3%)	20761 (54.3%)
Married	64617 (68.5%)	65965 (70.0%)	66103 (70.1%)	65784 (69.8%)	65083 (69.0%)	47731 (85.1%)	48439 (86.4%)	48568 (86.6%)	48314 (86.2%)	47635 (85.0%)	16886 (44.2%)	17526 (45.9%)	17535 (45.9%)	17470 (45.7%)	17448 (45.7%)
**Current BMI (kg/m^2)**															
<25kg/m2	31163 (33.1%)	31488 (33.4%)	32256 (34.2%)	33757 (35.8%)	35193 (37.3%)	15863 (28.3%)	16164 (28.8%)	16242 (29.0%)	16509 (29.4%)	16570 (29.6%)	15300 (40.0%)	15324 (40.1%)	16014 (41.9%)	17248 (45.1%)	18623 (48.7%)
25-<30 kg/m2	40034 (42.5%)	40092 (42.5%)	39751 (42.2%)	38891 (41.3%)	37724 (40.0%)	27755 (49.5%)	27697 (49.4%)	27375 (48.8%)	26928 (48.0%)	26394 (47.1%)	12279 (32.1%)	12395 (32.4%)	12376 (32.4%)	11963 (31.3%)	11330 (29.7%)

© 2025 Zouiouich S et al. *JAMA Network Open*.

>=30 kg/m2	20558 (21.8%)	20545 (21.8%)	20131 (21.4%)	19514 (20.7%)	19250 (20.4%)	11290 (20.1%)	11255 (20.1%)	11481 (20.5%)	11664 (20.8%)	12049 (21.5%)	9268 (24.3%)	9290 (24.3%)	8650 (22.6%)	7850 (20.5%)	7201 (18.8%)
Missing	2527 (2.7%)	2152 (2.3%)	2143 (2.3%)	2116 (2.2%)	2111 (2.2%)	1163 (2.1%)	953 (1.7%)	972 (1.7%)	968 (1.7%)	1056 (1.9%)	1364 (3.6%)	1199 (3.1%)	1171 (3.1%)	1148 (3.0%)	1055 (2.8%)
**First degree relative with cancer (%)**															
No	44250 (46.9%)	43766 (46.4%)	43448 (46.1%)	43368 (46.0%)	43361 (46.0%)	27268 (48.6%)	26846 (47.9%)	26802 (47.8%)	26571 (47.4%)	26718 (47.7%)	16982 (44.4%)	16920 (44.3%)	16646 (43.6%)	16797 (44.0%)	16643 (43.6%)
Yes	44709 (47.4%)	45817 (48.6%)	46183 (49.0%)	46142 (48.9%)	46122 (48.9%)	25585 (45.6%)	26398 (47.1%)	26519 (47.3%)	26590 (47.4%)	26393 (47.1%)	19124 (50.0%)	19419 (50.8%)	19664 (51.5%)	19552 (51.2%)	19729 (51.6%)
Missing	5323 (5.6%)	4694 (5.0%)	4650 (4.9%)	4768 (5.1%)	4795 (5.1%)	3218 (5.7%)	2825 (5.0%)	2749 (4.9%)	2908 (5.2%)	2958 (5.3%)	2105 (5.5%)	1869 (4.9%)	1901 (5.0%)	1860 (4.9%)	1837 (4.8%)
**Smoking status and dose**															
Never smoked	31660 (33.6%)	32977 (35.0%)	33617 (35.7%)	34319 (36.4%)	34936 (37.1%)	15750 (28.1%)	16237 (29.0%)	16530 (29.5%)	16848 (30.0%)	17408 (31.0%)	15910 (41.6%)	16740 (43.8%)	17087 (44.7%)	17471 (45.7%)	17528 (45.9%)
Quit, <= 20 cigs/day	25733 (27.3%)	26386 (28.0%)	26507 (28.1%)	26646 (28.3%)	26109 (27.7%)	16389 (29.2%)	16463 (29.4%)	16288 (29.0%)	16252 (29.0%)	15496 (27.6%)	9344 (24.5%)	9923 (26.0%)	10219 (26.7%)	10394 (27.2%)	10613 (27.8%)

© 2025 Zouiouich S et al. *JAMA Network Open*.

Quit, > 20 cigs/day	19570 (20.8%)	20120 (21.3%)	19986 (21.2%)	19845 (21.0%)	20150 (21.4%)	15258 (27.2%)	15686 (28.0%)	15590 (27.8%)	15340 (27.4%)	15294 (27.3%)	4312 (11.3%)	4434 (11.6%)	4396 (11.5%)	4505 (11.8%)	4856 (12.7%)
Currently smoking, <= 20 cigs/day	8807 (9.3%)	7515 (8.0%)	7023 (7.4%)	6463 (6.9%)	6039 (6.4%)	3632 (6.5%)	3263 (5.8%)	3262 (5.8%)	3097 (5.5%)	3189 (5.7%)	5175 (13.5%)	4252 (11.1%)	3761 (9.8%)	3366 (8.8%)	2850 (7.5%)
Currently smoking, > 20 cigs/day	4452 (4.7%)	3927 (4.2%)	3733 (4.0%)	3606 (3.8%)	3463 (3.7%)	2503 (4.5%)	2354 (4.2%)	2340 (4.2%)	2410 (4.3%)	2458 (4.4%)	1949 (5.1%)	1573 (4.1%)	1393 (3.6%)	1196 (3.1%)	1005 (2.6%)
Missing	4060 (4.3%)	3352 (3.6%)	3415 (3.6%)	3399 (3.6%)	3581 (3.8%)	2539 (4.5%)	2066 (3.7%)	2060 (3.7%)	2122 (3.8%)	2224 (4.0%)	1521 (4.0%)	1286 (3.4%)	1355 (3.5%)	1277 (3.3%)	1357 (3.6%)
**Frequency of physical activity (%)**															
Never or rarely	21476 (22.8%)	17381 (18.4%)	15555 (16.5%)	14175 (15.0%)	12942 (13.7%)	10272 (18.3%)	8186 (14.6%)	7502 (13.4%)	7146 (12.7%)	6902 (12.3%)	11204 (29.3%)	9195 (24.1%)	8053 (21.1%)	7029 (18.4%)	6040 (15.8%)
1-3 times per month	15617 (16.6%)	13721 (14.6%)	12676 (13.4%)	11636 (12.3%)	10471 (11.1%)	9031 (16.1%)	7803 (13.9%)	7247 (12.9%)	6575 (11.7%)	6042 (10.8%)	6586 (17.2%)	5918 (15.5%)	5429 (14.2%)	5061 (13.2%)	4429 (11.6%)
1-2 times per week	20582 (21.8%)	21335 (22.6%)	21059 (22.3%)	20301 (21.5%)	18920 (20.1%)	12768 (22.8%)	12995 (23.2%)	12604 (22.5%)	12139 (21.7%)	11331 (20.2%)	7814 (20.4%)	8340 (21.8%)	8455 (22.1%)	8162 (21.4%)	7589 (19.9%)
3-4 times per week	21698 (23.0%)	24849 (26.4%)	26043 (27.6%)	26961 (28.6%)	27706 (29.4%)	14115 (25.2%)	15812 (28.2%)	16319 (29.1%)	16480 (29.4%)	16415 (29.3%)	7583 (19.8%)	9037 (23.7%)	9724 (25.4%)	10481 (27.4%)	11291 (29.6%)

© 2025 Zouiouich S et al. *JAMA Network Open*.

5 or more times per week	13417 (14.2%)	15975 (16.9%)	18066 (19.2%)	20372 (21.6%)	23464 (24.9%)	9109 (16.2%)	10799 (19.3%)	11966 (21.3%)	13306 (23.7%)	14946 (26.7%)	4308 (11.3%)	5176 (13.5%)	6100 (16.0%)	7066 (18.5%)	8518 (22.3%)
Missing	1492 (1.6%)	1016 (1.1%)	882 (0.9%)	833 (0.9%)	775 (0.8%)	776 (1.4%)	474 (0.8%)	432 (0.8%)	423 (0.8%)	433 (0.8%)	716 (1.9%)	542 (1.4%)	450 (1.2%)	410 (1.1%)	342 (0.9%)
**History of diabetes (%)**															
No	86472 (91.7%)	86246 (91.5%)	86132 (91.4%)	86060 (91.3%)	85988 (91.2%)	51125 (91.2%)	50901 (90.8%)	50684 (90.4%)	50384 (89.9%)	49995 (89.2%)	35347 (92.5%)	35345 (92.5%)	35448 (92.8%)	35676 (93.4%)	35993 (94.2%)
Yes	7810 (8.3%)	8031 (8.5%)	8149 (8.6%)	8218 (8.7%)	8290 (8.8%)	4946 (8.8%)	5168 (9.2%)	5386 (9.6%)	5685 (10.1%)	6074 (10.8%)	2864 (7.5%)	2863 (7.5%)	2763 (7.2%)	2533 (6.6%)	2216 (5.8%)
**Current HRT use (women only, %)**															
Non-user	20625 (21.9%)	19172 (20.3%)	17875 (19.0%)	16360 (17.4%)	14858 (15.8%)	0 (0%)	0 (0%)	0 (0%)	0 (0%)	0 (0%)	20625 (54.0%)	19172 (50.2%)	17875 (46.8%)	16360 (42.8%)	14858 (38.9%)
User	17586 (18.7%)	19036 (20.2%)	20336 (21.6%)	21849 (23.2%)	23351 (24.8%)	0 (0%)	0 (0%)	0 (0%)	0 (0%)	0 (0%)	17586 (46.0%)	19036 (49.8%)	20336 (53.2%)	21849 (57.2%)	23351 (61.1%)
Male	56071 (59.5%)	56069 (59.5%)	56070 (59.5%)	56069 (59.5%)	56069 (59.5%)	56071 (100%)	56069 (100%)	56070 (100%)	56069 (100%)	56069 (100%)	0 (0%)	0 (0%)	0 (0%)	0 (0%)	0 (0%)

© 2025 Zouiouich S et al. *JAMA Network Open*.

**Frequency of multivitamin use (%)**															
Never	65167 (69.1%)	48251 (51.2%)	38908 (41.3%)	31914 (33.9%)	26461 (28.1%)	39740 (70.9%)	30440 (54.3%)	25524 (45.5%)	21343 (38.1%)	18099 (32.3%)	25427 (66.5%)	17811 (46.6%)	13384 (35.0%)	10571 (27.7%)	8362 (21.9%)
1-6 times per week	11065 (11.7%)	13267 (14.1%)	12873 (13.7%)	11392 (12.1%)	8628 (9.2%)	5787 (10.3%)	6655 (11.9%)	6811 (12.1%)	6627 (11.8%)	5436 (9.7%)	5278 (13.8%)	6612 (17.3%)	6062 (15.9%)	4765 (12.5%)	3192 (8.4%)
1 per day	16625 (17.6%)	29848 (31.7%)	38067 (40.4%)	44752 (47.5%)	50249 (53.3%)	9727 (17.3%)	17358 (31.0%)	21452 (38.3%)	24718 (44.1%)	27546 (49.1%)	6898 (18.1%)	12490 (32.7%)	16615 (43.5%)	20034 (52.4%)	22703 (59.4%)
>1 per day	1425 (1.5%)	2911 (3.1%)	4433 (4.7%)	6220 (6.6%)	8940 (9.5%)	817 (1.5%)	1616 (2.9%)	2283 (4.1%)	3381 (6.0%)	4988 (8.9%)	608 (1.6%)	1295 (3.4%)	2150 (5.6%)	2839 (7.4%)	3952 (10.3%)
**Alcohol consumption, g/day (%)**															
0	23678 (25.1%)	21725 (23.0%)	21387 (22.7%)	22194 (23.5%)	24183 (25.7%)	11601 (20.7%)	10681 (19.0%)	10731 (19.1%)	11432 (20.4%)	13112 (23.4%)	12077 (31.6%)	11044 (28.9%)	10656 (27.9%)	10762 (28.2%)	11071 (29.0%)
< 5 g/day	37542 (39.8%)	37442 (39.7%)	37290 (39.6%)	36910 (39.2%)	36609 (38.8%)	20036 (35.7%)	19221 (34.3%)	18854 (33.6%)	18827 (33.6%)	18876 (33.7%)	17506 (45.8%)	18221 (47.7%)	18436 (48.2%)	18083 (47.3%)	17733 (46.4%)
5 - < 15 g/day	13867	14961	15230	15006	14349	9588 (17.1%)	10236	10295	9911 (17.7%)	9192 (16.4%)	4279 (11.2%)	4725 (12.4%)	4935 (12.9%)	5095 (13.3%)	5157 (13.5%)

© 2025 Zouiouich S et al. *JAMA Network Open*.

	(14.7%)	(15.9%)	(16.2%)	(15.9%)	(15.2%)		(18.3%)	(18.4%)							
15 - < 30 g/day	9649 (10.2%)	10456 (11.1%)	10409 (11.0%)	10337 (11.0%)	9797 (10.4%)	7265 (13.0%)	7998 (14.3%)	7988 (14.2%)	7755 (13.8%)	7225 (12.9%)	2384 (6.2%)	2458 (6.4%)	2421 (6.3%)	2582 (6.8%)	2572 (6.7%)
>= 30 g/day	9546 (10.1%)	9693 (10.3%)	9965 (10.6%)	9831 (10.4%)	9340 (9.9%)	7581 (13.5%)	7933 (14.1%)	8202 (14.6%)	8144 (14.5%)	7664 (13.7%)	1965 (5.1%)	1760 (4.6%)	1763 (4.6%)	1687 (4.4%)	1676 (4.4%)
**Alcohol consumption, g/day (%)**															
Mean (SD)	11.4 (28.8)	12.0 (28.9)	12.8 (32.4)	12.9 (34.2)	12.0 (31.6)	15.1 (33.8)	16.0 (33.8)	17.4 (38.7)	17.7 (41.5)	16.3 (38.3)	6.09 (17.9)	5.99 (17.9)	5.96 (17.4)	5.90 (16.5)	5.76 (15.6)
Median [Min, Max]	1.65 [0, 685]	1.94 [0, 638]	2.01 [0, 500]	1.97 [0, 631]	1.73 [0, 526]	3.21 [0, 685]	3.99 [0, 638]	4.11 [0, 486]	3.78 [0, 631]	3.11 [0, 482]	0.740 [0, 501]	0.860 [0, 535]	0.980 [0, 500]	1.00 [0, 498]	0.980 [0, 526]
**Whole grains (servings/1000 kcal/day)**															
Mean (SD)	0.509 (0.437)	0.573 (0.428)	0.584 (0.419)	0.595 (0.418)	0.595 (0.414)	0.505 (0.440)	0.574 (0.435)	0.583 (0.427)	0.590 (0.425)	0.581 (0.418)	0.515 (0.432)	0.572 (0.416)	0.586 (0.408)	0.602 (0.406)	0.614 (0.407)
Median [Min, Max]	0.398 [0, 6.99]	0.482 [0, 5.77]	0.501 [0, 5.33]	0.512 [0, 4.97]	0.513 [0, 4.80]	0.392 [0, 5.02]	0.481 [0, 5.77]	0.501 [0, 5.33]	0.505 [0, 4.70]	0.496 [0, 4.51]	0.406 [0, 6.99]	0.484 [0, 5.01]	0.502 [0, 4.77]	0.521 [0, 4.97]	0.536 [0, 4.80]
**Fruit and vegetable intake (servings/day)**															

© 2025 Zouiouich S et al. *JAMA Network Open*.

Mean (SD)	2.25 (1.15)	2.28 (1.08)	2.30 (1.08)	2.32 (1.09)	2.36 (1.15)	2.11 (1.07)	2.11 (0.992)	2.11 (0.979)	2.12 (0.991)	2.11 (1.05)	2.46 (1.22)	2.52 (1.16)	2.59 (1.16)	2.63 (1.15)	2.72 (1.20)
Median [Min, Max]	2.05 [0.0661, 13.4]	2.10 [0.0571, 12.3]	2.12 [0.0111, 13.0]	2.15 [0.0631, 12.1]	2.17 [0.0307, 14.6]	1.91 [0.0661, 12.3]	1.95 [0.0571, 11.5]	1.95 [0.0111, 11.8]	1.96 [0.0631, 12.1]	1.93 [0.0307, 11.8]	2.26 [0.103, 13.4]	2.34 [0.0626, 12.3]	2.41 [0.0822, 13.0]	2.46 [0.0669, 11.8]	2.54 [0.0442, 14.6]
**Red meat, not processed (g/1000 kcal/day)**															
Mean (SD)	29.0 (19.1)	27.0 (17.5)	25.9 (16.9)	24.6 (16.5)	22.2 (15.8)	30.8 (19.7)	29.0 (18.0)	28.0 (17.4)	26.8 (17.0)	24.5 (16.4)	26.4 (17.8)	24.1 (16.2)	22.9 (15.7)	21.3 (15.1)	18.9 (14.4)
Median [Min, Max]	25.6 [0, 224]	24.0 [0, 247]	23.0 [0, 222]	21.8 [0, 239]	19.2 [0, 205]	27.4 [0, 224]	26.0 [0, 189]	25.2 [0, 222]	24.1 [0, 239]	21.6 [0, 190]	23.1 [0, 177]	21.3 [0, 247]	20.0 [0, 186]	18.5 [0, 237]	16.0 [0, 205]
**Processed meat (g/1000 kcal/day)**															
Mean (SD)	11.4 (11.4)	11.0 (10.3)	10.6 (9.92)	10.1 (9.72)	9.12 (9.13)	12.8 (12.1)	12.5 (10.8)	12.2 (10.5)	11.7 (10.3)	10.6 (9.66)	9.40 (10.0)	8.79 (8.88)	8.25 (8.41)	7.81 (8.29)	6.97 (7.82)
Median [Min, Max]	8.14 [0, 256]	8.10 [0, 187]	7.83 [0, 164]	7.37 [0, 210]	6.47 [0, 199]	9.40 [0, 256]	9.56 [0, 140]	9.53 [0, 164]	9.03 [0, 210]	8.03 [0, 199]	6.52 [0, 218]	6.24 [0, 187]	5.82 [0, 131]	5.34 [0, 189]	4.56 [0, 151]
**Supplemental folate (ug/day)**															
Mean (SD)	100 (178)	175 (214)	225 (229)	268 (240)	313 (258)	97.1 (177)	168 (215)	209 (228)	249 (242)	293 (263)	105 (179)	186 (213)	248 (230)	295 (235)	341 (246)

© 2025 Zouiouich S et al. *JAMA Network Open*.

Median [Min, Max]	0 [0, 1200]	0 [0, 1200]	286 [0, 1200]	400 [0, 1200]	400 [0, 1200]	0 [0, 1200]	0 [0, 1200]	114 [0, 1200]	400 [0, 1200]	400 [0, 1200]	0 [0, 1200]	57.2 [0, 1200]	286 [0, 1200]	400 [0, 1200]	400 [0, 1200]
**Vitamin D (ug/1000 kcal/day)**															
Mean (SD)	1.83 (0.956)	2.18 (1.06)	2.43 (1.20)	2.74 (1.39)	3.48 (1.88)	1.81 (0.923)	2.11 (0.985)	2.36 (1.10)	2.67 (1.25)	3.61 (1.88)	1.86 (1.00)	2.28 (1.15)	2.52 (1.32)	2.84 (1.56)	3.29 (1.86)
Median [Min, Max]	1.64 [0, 19.0]	2.02 [0.0530, 17.5]	2.24 [0.0289, 15.9]	2.51 [0.0129, 14.9]	3.14 [0.0115, 17.8]	1.63 [0, 11.1]	1.97 [0.0676, 17.5]	2.20 [0.0289, 14.7]	2.49 [0.0693, 13.0]	3.28 [0.0115, 17.8]	1.66 [0.0170, 19.0]	2.09 [0.0530, 14.6]	2.30 [0.0642, 15.9]	2.54 [0.0129, 14.9]	2.92 [0.0120, 16.8]
**Vitamin C (mg/1000 kcal/day)**															
Mean (SD)	87.5 (57.3)	89.8 (53.9)	91.8 (54.1)	93.4 (54.3)	95.9 (55.8)	82.6 (54.8)	83.8 (50.9)	84.5 (50.5)	85.8 (51.1)	87.3 (52.7)	94.8 (60.0)	98.8 (57.0)	102 (57.2)	105 (56.8)	109 (57.9)
Median [Min, Max]	74.5 [1.88, 747]	78.9 [1.48, 719]	80.9 [0.336, 719]	82.6 [2.21, 751]	84.6 [2.59, 692]	69.9 [1.88, 747]	73.4 [1.48, 593]	74.3 [0.336, 719]	75.5 [2.21, 652]	75.8 [2.59, 580]	81.7 [2.10, 712]	87.5 [2.57, 719]	91.7 [3.37, 661]	94.3 [2.64, 751]	97.4 [3.46, 692]
**Total energy (kcal/day)**															
Mean (SD)	1260 (467)	1630 (543)	1850 (666)	2050 (790)	2360 (962)	1350 (485)	1750 (541)	2010 (654)	2260 (774)	2630 (948)	1140 (406)	1470 (503)	1610 (609)	1730 (702)	1950 (829)
Median [Min, Max]	1210 [342, 5860]	1570 [342, 5810]	1760 [347, 5870]	1930 [350, 5880]	2180 [343, 5880]	1300 [342, 5860]	1680 [351, 5810]	1930 [389, 5870]	2140 [373, 5880]	2470 [360, 5880]	1090 [342, 4970]	1410 [342, 5580]	1520 [347, 5810]	1610 [350, 5840]	1780 [343, 5870]

© 2025 Zouiouich S et al. *JAMA Network Open*.

eTable 2. Distribution of Calcium Intake by Sex-Specific Quintiles in Each Calcium Source for Males and Females

Distribution of calcium intake by sex-specific quintiles in each calcium source for males (N=280,348) and females (N=191,048). Calcium intake was estimated from diet only, including dairy and non-dairy calcium (mg/day). Calcium intake from supplements (i.e, supplemental calcium, mg/day) was estimated from multivitamins and calcium supplements and categorized into four groups, i.e., 0 mg/day, >0–<400 mg/day, 400–<1,000 mg/day, and ≥1,000 mg/day. Total calcium intake, in mg/day, was calculated as the sum of dietary calcium (mg/day) intake and supplemental calcium intake (mg/day).

	**Male**									
	**Quintile 1**		**Quintile 2**		**Quintile 3**		**Quintile 4**		**Quintile 5**	
**Calcium Source**	Min	Max	Min	Maxi	Min	Max	Min	Max	Min	Max
Total Calcium	105.32	537.55	537.56	727.67	727.68	944.02	944.04	1287.42	1287.44	4939.35
Dietary Calcium	105.31	462.82	462.83	619.52	619.53	795.02	795.03	1073.25	1073.26	3468.26
Dairy Calcium	0	159.97	159.98	273.27	273.28	407.89	407.9	646.33	646.34	3315.75
Non-Dairy Calcium	18.6	237.66	237.67	302.4	302.41	369.76	369.77	467.87	467.88	2509.17
Supplemental Calcium	0	0	23.1	380.4	401.5	1000	1023.1	1662		
	**Female**									
	**Quintile 1**		**Quintile 2**		**Quintile 3**		**Quintile 4**		**Quintile 5**	
**Calcium Source**	Min	Max	Min	Maxi	Min	Max	Min	Max	Min	Max
Total Calcium	105.68	554.78	554.8	821.26	821.27	1132.42	1132.43	1586.28	1586.29	5010.49
Dietary Calcium	105.6	395.08	395.09	541.65	541.66	707.17	707.18	971.74	971.75	3466.34
Dairy Calcium	0	133.76	133.77	242.4	242.41	373.63	373.64	593.01	593.03	3270.16
Non-Dairy Calcium	26.44	201.55	201.56	259.1	259.11	319.66	319.67	408.87	408.88	2171.18
Supplemental Calcium	0	0	23.1	380.4	401.5	1000	1023.1	1662		

© 2025 Zouiouich S et al. *JAMA Network Open*.

eTable 3. Adjusted Associations of Colorectal Cancer Incidence With Sex-Specific Quintiles of Total, Dietary, Dairy, and Nondairy Calcium and Categories of Supplemental Calcium in the NIH-AARP Diet and Health Study

	**Both sex**					
	**Quintile 1**	**Quintile 2**	**Quintile 3**	**Quintile 4**	**Quintile 5**	**p trend**
**Total calcium**[a]						
HR (95% CI)	1	0.93 (0.88-0.99)	0.82 (0.77-0.88)	0.76 (0.71-0.82)	0.71 (0.65-0.78)	<0.001
No. of cases	2444	2320	2077	1940	1837	
**Dietary calcium**[b]						
HR (95% CI)	1	0.88 (0.83-0.93)	0.85 (0.80-0.91)	0.85 (0.80-0.91)	0.84 (0.77-0.92)	0.001
No. of cases	2566	2180	2041	1979	1852	
**Dairy calcium**[c]						
HR (95% CI)	1	0.97 (0.91-1.03)	0.90 (0.84-0.96)	0.84 (0.78-0.90)	0.81 (0.74-0.89)	<0.001
No. of cases	2335	2280	2113	1984	1906	
**Non-dairy calcium**[d]						
HR (95% CI)	1	0.90 (0.84-0.95)	0.93 (0.87-0.99)	0.89 (0.83-0.96)	0.87 (0.79-0.96)	0.02
No. of cases	2222	2022	2123	2082	2169	
**Supplemental calcium**[e]						
HR (95% CI)	1	0.99 (0.93-1.05)	0.86 (0.80-0.92)	0.80 (0.72-0.90)	-	<0.001
No. of cases	4951	3718	1508	441	-	

[a] In mg/day. Multivariable cox proportional hazards model adjusted for the following: Sex, race, baseline age, education, marital status, BMI, family history of cancer, smoking dose and status, physical activity, frequency of multivitamin use, alcohol intake, whole grains, fruit and vegetable, red unprocessed meat, processed meat, supplemental folate, vitamin D, vitamin C, and total energy intake per day.

[b] In mg/1000 kcal/day. Adjusted for same covariates in Model A + supplemental calcium intake.

[c] In mg/day. Adjusted for same covariates in Model B + calcium from non-dairy sources

[d] In mg/day. Adjusted for same covariates in Model B + calcium from dairy sources.

© 2025 Zouiouich S et al. *JAMA Network Open*.

[e] In mg/day. Adjusted for same covariates in Model B + calcium from dairy and non-dairy sources. Supplemental calcium results were categorized into 4 groups: group 1 (G1), 0mg/d; G2, more than 0 to less than 400mg/d; G3, 400 to less than 1000mg/day; G4, 1000mg/d or more.

© 2025 Zouiouich S et al. *JAMA Network Open*.

eTable 4. Adjusted Associations of Colorectal Cancer Incidence With Sex-Specific Quintiles of Total, Dietary, Dairy, and Nondairy Calcium and Categories of Supplemental Calcium by Sex in the NIH-AARP Diet and Health Study

	**Female**						**Male**					
	**Quintile 1**	**Quintile 2**	**Quintile 3**	**Quintile 4**	**Quintile 5**	**p trend**	**Quintile 1**	**Quintile 2**	**Quintile 3**	**Quintile 4**	**Quintile 5**	**p trend**
**Total calcium[a]**												
HR (95% CI)	1	0.88 (0.80-0.98)	0.79 (0.71-0.88)	0.72 (0.64-0.82)	0.71 (0.62-0.81)	<0.001	1	0.96 (0.89-1.03)	0.84 (0.77-0.91)	0.78 (0.70-0.85)	0.71 (0.63-0.80)	<0.001
No. of cases	920	823	737	679	663		1524	1497	1340	1261	1174	
**Dietary calcium[b]**												
HR (95% CI)	1	0.89 (0.81-0.99)	0.89 (0.80-0.99)	0.82 (0.73-0.92)	0.79 (0.68-0.92)	0.002	1	0.87 (0.81-0.94)	0.83 (0.77-0.90)	0.88 (0.80-0.96)	0.87 (0.77-0.98)	0.07
No. of cases	891	790	774	698	669		1675	1390	1267	1281	1183	
**Dairy calcium[c]**												
HR (95% CI)	1	1.00 (0.91-1.11)	0.89 (0.80-0.99)	0.82 (0.73-0.93)	0.75 (0.64-0.88)	<0.001	1	0.95 (0.88-1.02)	0.90 (0.83-0.98)	0.85 (0.78-0.93)	0.84 (0.74-0.94)	0.003
No. of cases	822	844	760	717	679		1513	1436	1353	1267	1227	
**Non-dairy calcium[d]**												
HR (95% CI)	1	0.93 (0.84-1.03)	0.96 (0.86-1.07)	0.90 (0.80-1.02)	0.90 (0.76-1.05)	0.21	1	0.88 (0.81-0.95)	0.91 (0.83-0.99)	0.88 (0.80-0.97)	0.86 (0.76-0.97)	0.05

© 2025 Zouiouich S et al. *JAMA Network Open*.

No. of cases	785	736	773	742	786		1437	1286	1350	1340	1383	
**Supplemental calcium[e]**											
HR (95% CI)	1	0.97 (0.88-1.08)	0.85 (0.77-0.94)	0.80 (0.70-0.92)	-	<0.001	1	0.99 (0.92-1.07)	0.85 (0.77-0.94)	0.79 (0.64-0.97)	-	<0.001
No. of cases	1345	1206	930	341	-		3606	2512	578	100	-	

[a] In mg/day. Multivariable cox proportional hazards model adjusted for the following: Sex, race, baseline age, education, marital status, BMI, family history of cancer, smoking dose and status, physical activity, frequency of multivitamin use, alcohol intake, whole grains, fruit and vegetable, red unprocessed meat, processed meat, supplemental folate, vitamin D, vitamin C, and total energy intake per day.

[b] In mg/1000 kcal/day. Adjusted for same covariates in Model A + supplemental calcium intake.

[c] In mg/day. Adjusted for same covariates in Model B + calcium from non-dairy sources

[d] In mg/day. Adjusted for same covariates in Model B + calcium from dairy sources.

[e] In mg/day. Adjusted for same covariates in Model B + calcium from dairy and non-dairy sources. Supplemental calcium results were categorized into 4 groups: group 1 (G1), 0mg/d; G2, more than 0 to less than 400mg/d; G3, 400 to less than 1000mg/day; G4, 1000mg/d or more.

© 2025 Zouiouich S et al. *JAMA Network Open*.

eTable 5. Adjusted Associations of Colorectal Cancer Incidence With Continuous Total, Dietary, Dairy, and Nondairy, Supplemental Calcium by Race and Ethnicity in the NIH-AARP Diet and Health Study

	**All participants**		**Non-Hispanic White participants**		**Non-Hispanic Black participants**		**Hispanic participants**		**Asian, Pacific Islander, Native American participants**
	Continuous - 300 mg/day increase		*Continuous - 300 mg/day increase*		*Continuous - 300 mg/day increase*		*Continuous - 300 mg/day increase*		*Continuous - 300 mg/day increase*
**Total calcium**[a]									
HR (95% CI)	0.92 (0.90-0.95)		0.93 (0.90-0.95)		0.68 (0.56-0.82)		0.83 (0.67-1.02)		0.94 (0.76-1.16)
**Dietary calcium**[b]									
HR (95% CI)	0.90 (0.84-0.96)		0.92 (0.86-0.99)		0.64 (0.49-0.85)		0.81 (0.51-1.28)		0.83 (0.49-1.40)
**Dairy calcium**[c]									
HR (95% CI)	0.89 (0.83-0.95)		0.91 (0.85-0.98)		0.63 (0.47-0.83)		0.83 (0.52-1.33)		0.75 (0.44-1.30)
**Non-dairy calcium**[d]									
HR (95% CI)	0.93 (0.80-1.08)		0.94 (0.80-1.10)		0.73 (0.39-1.36)		0.71 (0.26-1.92)		1.22 (0.46-3.24)
**Supplemental calcium**[e]									
HR (95% CI)	0.95 (0.93-0.97)		0.95 (0.93-0.97)		0.81 (0.69-0.96)		0.91 (0.76-1.08)		0.92 (0.76-1.11)

[a] In mg/day. Multivariable cox proportional hazards model adjusted for the following: Sex, baseline age, education, marital status, BMI, family history of cancer, smoking dose and status, physical activity, frequency of multivitamin use, alcohol intake, whole grains, fruit and vegetable, red unprocessed meat, processed meat, supplemental folate, vitamin D, vitamin C, and total energy intake per day.

© 2025 Zouiouich S et al. *JAMA Network Open*.

[b] In mg/1000 kcal/day. Adjusted for same covariates in Model A + supplemental calcium intake.
[c] In mg/day. Adjusted for same covariates in Model B + calcium from non-dairy sources
[d] In mg/day. Adjusted for same covariates in Model B + calcium from dairy sources.
[e] In mg/day. Adjusted for same covariates in Model B + calcium from dairy and non-dairy sources.

© 2025 Zouiouich S et al. *JAMA Network Open*.

eTable 6. Adjusted Associations of Colorectal Cancer Incidence With Quintiles of Total Calcium by Tumor Site of Colon and Rectum in the NIH-AARP Diet and Health Study

	**Total calcium**					
	**Quintile 1**	**Quintile 2**	**Quintile 3**	**Quintile 4**	**Quintile 5**	**p trend**
**Proximal**	1	0.93 (0.86-1.02)	0.82 (0.74-0.90)	0.76 (0.68-0.84)	0.75 (0.66-0.86)	<0.001
No. of cases	1135	1082	961	896	894	
**Cecum**	1	0.96 (0.84-1.11)	0.85 (0.73-0.99)	0.79 (0.67-0.94)	0.86 (0.71-1.04)	0.07
No. of cases	463	442	387	356	372	
**Ascending colon**	1	0.86 (0.74-1.00)	0.81 (0.69-0.96)	0.76 (0.63-0.91)	0.71 (0.57-0.88)	0.007
No. of cases	392	351	341	325	316	
**Hepatic flexure**	1	1.11 (0.84-1.46)	0.82 (0.60-1.12)	0.64 (0.45-0.92)	0.59 (0.39-0.91)	0.003
No. of cases	108	125	96	78	75	
**Transverse colon**	1	0.91 (0.73-1.15)	0.74 (0.57-0.95)	0.71 (0.54-0.94)	0.64 (0.46-0.89)	0.02
No. of cases	172	164	137	137	131	
**Distal**	1	0.99 (0.88-1.11)	0.88 (0.78-1.00)	0.73 (0.63-0.84)	0.73 (0.61-0.87)	<0.001
No. of cases	654	644	575	476	472	
**Splenic flexure**	1	0.98 (0.68-1.40)	0.91 (0.61-1.35)	0.64 (0.40-1.03)	0.53 (0.29-0.95)	0.009
No. of cases	72	66	60	41	33	
**Descending colon**	1	0.90 (0.67-1.21)	0.93 (0.68-1.27)	0.68 (0.47-0.98)	0.51 (0.32-0.80)	<0.001
No. of cases	102	98	106	81	64	
**Sigmoid colon**	1	1.01 (0.89-1.16)	0.86 (0.74-1.00)	0.75 (0.63-0.89)	0.80 (0.66-0.98)	0.01
No. of cases	480	480	409	354	375	
**Rectum**	1	0.87 (0.77-0.98)	0.76 (0.66-0.87)	0.77 (0.66-0.89)	0.61 (0.51-0.74)	<0.001
No. of cases	600	540	485	503	418	
**Rectosigmoid junction**	1	0.90 (0.72-1.13)	0.83 (0.65-1.06)	0.81 (0.62-1.07)	0.62 (0.44-0.87)	0.01
No. of cases	176	160	151	151	119	
**Rectum**	1	0.86 (0.75-1.00)	0.73 (0.62-0.86)	0.74 (0.62-0.89)	0.60 (0.49-0.75)	<0.001

© 2025 Zouiouich S et al. *JAMA Network Open*.

No. of cases	424	380	334	352	299	
In mg/day. Multivariable cox proportional hazards model adjusted for the following: Sex, race, baseline age, education, marital status, BMI, family history of cancer, smoking dose and status, physical activity, frequency of multivitamin use, alcohol intake, whole grains, fruit and vegetable, red unprocessed meat, processed meat, supplemental folate, vitamin D, vitamin C, and total energy intake per day.						

© 2025 Zouiouich S et al. *JAMA Network Open*.

eTable 7. Adjusted Associations of Colorectal Cancer Incidence With Quintiles of Dietary Calcium by Tumor Site of Colon and Rectum in the NIH-AARP Diet and Health Study

	**Dietary calcium**					
	**Quintile 1**	**Quintile 2**	**Quintile 3**	**Quintile 4**	**Quintile 5**	**p trend**
**Proximal**	1	0.88 (0.80- 0.96)	0.86 (0.78-0.94)	0.90 (0.81-0.99)	0.87 (0.76-1.00)	0.11
No. of cases	1148	995	950	971	904	
**Cecum**	1	0.96 (0.84-1.10)	0.87 (0.75-1.01)	0.93 (0.792-1.09)	0.96 (0.78-1.19)	0.53
No. of cases	455	434	382	389	360	
**Ascending colon**	1	0.81 (0.69-0.94)	0.84 (0.72-0.98)	0.86 (0.72-1.02)	0.80 (0.64-1.01)	0.19
No. of cases	402	325	334	340	324	
**Hepatic flexure**	1	0.98 (0.74-1.30)	0.99 (0.73-1.33)	1.01 (0.73-1.40)	0.71 (0.45-1.12)	0.22
No. of cases	103	101	101	103	74	
**Transverse colon**	1	0.76 (0.60-0.95)	0.78 (0.61-0.99)	0.83 (0.64-1.08)	0.88 (0.62-1.23)	0.69
No. of cases	188	135	133	139	146	
**Distal**	1	0.85 (0.76-0.95)	0.80 (0.71-0.90)	0.80 (0.70-0.92)	0.73 (0.60-0.87)	0.002
No. of cases	725	594	535	520	447	
**Splenic flexure**	1	0.93 (0.66-1.31)	0.80 (0.55-1.18)	0.71 (0.45-1.10)	0.44 (0.23-0.84)	0.01
No. of cases	77	66	54	46	29	
**Descending colon**	1	0.72 (0.54-0.96)	0.73 (0.54-1.00)	0.82 (0.59-1.14)	0.64 (0.40-1.01)	0.16
No. of cases	117	86	86	93	69	
**Sigmoid colon**	1	0.87 (0.76-0.99)	0.81 (0.70-0.93)	0.81 (0.69-0.95)	0.78 (0.63-0.96)	0.04
No. of cases	531	442	395	381	349	
**Rectum**	1	0.93 (0.82-1.05)	0.93 (0.82-1.06)	0.83 (0.72-0.96)	0.90 (0.74-1.09)	0.19
No. of cases	624	535	509	435	443	
**Rectosigmoid junction**	1	0.90 (0.72-1.13)	1.01 (0.80-1.27)	0.78 (0.59-1.02)	1.00 (0.71-1.41)	0.89
No. of cases	183	153	161	118	142	
**Rectum**	1	0.93 (0.81-1.08)	0.89 (0.77-1.04)	0.85 (0.71-1.01)	0.84 (0.67-1.06)	0.11

© 2025 Zouiouich S et al. *JAMA Network Open*.

No. of cases	441	382	348	317	301	
In mg/1000 kcal/day. Multivariable cox proportional hazards model adjusted for the following: Sex, race, baseline age, education, marital status, BMI, family history of cancer, smoking dose and status, physical activity, frequency of multivitamin use, alcohol intake, whole grains, fruit and vegetable, red unprocessed meat, processed meat, supplemental folate, vitamin D, vitamin C, calcium supplements and total energy intake per day.						

© 2025 Zouiouich S et al. *JAMA Network Open*.

eTable 8. Adjusted Associations of Colorectal Cancer Incidence With Categories of Supplemental Calcium by Tumor Site of Colon and Rectum in the NIH-AARP Diet and Health Study

	**Supplemental Calcium**				
	**0**	**>0–<400**	**400–<1,000**	**≥1,000**	**p trend**
**Proximal**	1	0.97 (0.89-1.06)	0.84 (0.76-0.93)	0.87 (0.75-1.01)	0.001
No. of cases	2253	1714	751	250	
**Cecum**	1	0.97 (0.84-1.11)	0.87 (0.74-1.01)	0.90 (0.72-1.13)	0.63
No. of cases	901	688	321	110	
**Ascending colon**	1	0.95 (0.82-1.11)	0.83 (0.70-0.98)	0.92 (0.72-1.18)	0.10
No. of cases	771	600	260	94	
**Hepatic flexure**	1	1.03 (0.78-1.37)	0.82 (0.59-1.15)	0.65 (0.38-1.13)	0.04
No. of cases	216	181	68	17	
**Transverse colon**	1	0.94 (0.74-1.18)	0.80 (0.61-1.04)	0.73 (0.47-1.11)	0.06
No. of cases	365	245	102	29	
**Distal**	1	1.00 (0.89-1.12)	0.90 (0.78-1.03)	0.81 (0.65-1.01)	0.02
No. of cases	1365	966	387	103	
**Splenic flexure**	1	1.09 (0.75-1.60)	0.86 (0.55-1.34)	0.79 (0.38-1.62)	0.27
No. of cases	138	89	35	10	
**Descending colon**	1	0.97 (0.72-1.30)	0.86 (0.60-1.212	0.57 (0.30-1.07)	0.07
No. of cases	220	158	61	12	
**Sigmoid colon**	1	0.99 (0.86-1.13)	0.91 (0.77-1.06)	0.86 (0.67-1.12)	0.11
No. of cases	1007	719	291	81	
**Rectum**	1	1.03 (0.91-1.16)	0.81 (0.70-0.94)	0.65 (0.50-0.83)	0.01
No. of cases	1209	935	324	78	
**Rectosigmoid junction**	1	1.16 (0.92-1.46)	0.93 (0.71-1.23)	0.71 (0.45-1.14)	0.04
No. of cases	345	287	102	23	
**Rectum**	1	0.97 (0.84-1.12)	0.75 (0.63-0.90)	0.61 (0.45-0.83)	<0.001
No. of cases	864	648	222	55	

© 2025 Zouiouich S et al. *JAMA Network Open*.

In mg/1000 kcal/day. Multivariable cox proportional hazards model adjusted for the following: Sex, race, baseline age, education, marital status, BMI, family history of cancer, smoking dose and status, physical activity, frequency of multivitamin use, alcohol intake, whole grains, fruit and vegetable, red unprocessed meat, processed meat, supplemental folate, vitamin D, vitamin C and total energy intake per day.					

© 2025 Zouiouich S et al. *JAMA Network Open*.

eTable 9. Adjusted Associations of Colorectal Cancer Incidence With Quintiles of Total, Dietary, Dairy, and Nondairy Calcium and Categories of Supplemental Calcium in Participants Who Were American Indian or Alaska Native, Asian, or Pacific Islander; Hispanic; Non-Hispanic Black; or Non-Hispanic White in the NIH-AARP Diet and Health Study

	**Non-Hispanic White participants**						**Non-Hispanic Black participants**						**Hispanic participants**						**Asian, Pacific Islander, Native American participants**					
	**Quintile 1**	**Quintile 2**	**Quintile 3**	**Quintile 4**	**Quintile 5**	**p trend**	**Quintile 1**	**Quintile 2**	**Quintile 3**	**Quintile 4**	**Quintile 5**	**p trend**	**Quintile 1**	**Quintile 2**	**Quintile 3**	**Quintile 4**	**Quintile 5**	**p trend**	**Quintile 1**	**Quintile 2**	**Quintile 3**	**Quintile 4**	**Quintile 5**	**p trend**
**Total calcium[a]**																								
HR (95% CI)	1	0.94 (0.88-1.00)	0.83 (0.77-0.89)	0.76 (0.69-0.83)	0.73 (0.64-0.82)	<0.001	1	1.03 (0.77-1.37)	0.76 (0.53-1.10)	0.80 (0.51-1.25)	0.60 (0.32-1.13)	0.12	1	0.75 (0.48-1.18)	0.85 (0.52-1.40)	0.68 (0.37-1.26)	0.72 (0.30-1.72)	0.36	1	0.84 (0.50-1.41)	0.68 (0.36-1.27)	0.83 (0.40-1.70)	0.46 (0.16-1.32)	0.37
No. of cases	2169	2133	1921	1787	1719		125	100	67	65	44		57	35	39	34	34		43	29	22	29	17	
**Dietary**																								

© 2025 Zouiouich S et al. *JAMA Network Open*.

**calcium[b]**																								
HR (95% CI)	1	0.90 (0.84-0.95)	0.86 (0.80-0.92)	0.86 (0.80-0.93)	0.86 (0.78-0.95)	0.008	1	0.71 (0.53-0.94)	0.84 (0.62-1.14)	0.70 (0.49-1.00)	0.69 (0.44-1.09)	0.08	1	0.81 (0.52-1.26)	0.81 (0.51-1.27)	0.86 (0.53-1.40)	0.67 (0.35-1.26)	0.22	1	0.87 (0.55-1.40)	0.82 (0.48-1.40)	0.59 (0.30-1.13)	0.87 (0.41-1.86)	0.68
No. of cases	2275	2017	1881	1837	1719		146	75	73	55	52		49	35	38	40	37		52	30	23	15	20	
**Dairy calcium[c]**																								
HR (95% CI)	1	0.98 (0.92-1.05)	0.91 (0.85-0.97)	0.85 (0.78-0.91)	0.83 (0.76-0.92)	<0.001	1	0.81 (0.61-1.08)	0.88 (0.65-1.20)	0.84 (0.60-1.17)	0.50 (0.32-0.80)	0.008	1	0.71 (0.46-1.10)	0.86 (0.55-1.34)	0.82 (0.51-1.34)	0.58 (0.30-1.13)	0.21	1	0.92 (0.58-1.47)	0.58 (0.32-1.04)	0.92 (0.52-1.63)	0.43 (0.18-1.00)	0.07
No. of cases	2013	2119	1970	1836	1791		154	73	67	63	44		60	33	37	37	32		59	29	16	24	12	

© 2025 Zouiouich S et al. *JAMA Network Open*.

**Non-dairy calcium**[d]																								
HR (95% CI)	1	0.90 (0.84-0.96)	0.93 (0.87-1.00)	0.91 (0.84-0.98)	0.88 (0.79-0.97)	0.04	1	0.99 (0.70-1.40)	1.09 (0.77-1.55)	0.63 (0.42-0.95)	0.97 (0.63-1.48)	0.56	1	0.73 (0.45-1.19)	1.03 (0.64-1.67)	0.84 (0.49-1.43)	0.94 (0.48-1.84)	0.92	1	0.55 (0.30-1.03)	0.85 (0.48-1.51)	0.91 (0.50-1.66)	0.78 (0.37-1.66)	0.94
No. of cases	2019	1888	1983	1948	1891		72	60	67	49	153		49	28	37	33	52		36	15	23	26	40	
**Supplemental calcium**[e]																								
HR (95% CI)	1	1.00 (0.94-1.06)	0.87 (0.80-0.93)	0.81 (0.72-0.91)	-	<0.001	1	1.02 (0.75-1.38)	0.60 (0.39-0.92)	0.50 (0.18-1.39)	-	0.005	1	0.63 (0.41-0.	0.75 (0.46-1.	0.30 (0.09-1.	-	0.17	1	1.12 (0.67-1.	0.98 (0.54-1.	0.83 (0.30-2.	-	0.63

© 2025 Zouiouich S et al. *JAMA Network Open*.

														97)	23)	00)				88)	77)	29)		
No. of cases	4500	3409	1400	420	-		211	154	32	4	-		102	61	33	3	-		59	52	24	5	-	

a In mg/day. Multivariable cox proportional hazards model adjusted for the following: Sex, baseline age, education, marital status, BMI, family history of cancer, smoking dose and status, physical activity, frequency of multivitamin use, alcohol intake, whole grains, fruit and vegetable, red unprocessed meat, processed meat, supplemental folate, vitamin D, vitamin C, and total energy intake per day.

[b] In mg/1000 kcal/day. Adjusted for same covariates in Model A + supplemental calcium intake.

[c] In mg/day. Adjusted for same covariates in Model B + calcium from non-dairy sources

[d] In mg/day. Adjusted for same covariates in Model B + calcium from dairy sources.

[e] In mg/day. Adjusted for same covariates in Model B + calcium from dairy and non-dairy sources. Supplemental calcium results were categorized into 4 groups: group 1 (G1), 0mg/d; G2, more than 0 to less than 400mg/d; G3, 400 to less than 1000mg/day; G4, 1000mg/d or more.

© 2025 Zouiouich S et al. *JAMA Network Open*.
