# Supplementary material for: Calcium Intake and Risk of Colorectal Cancer in the NIH-AARP Diet and Health Study
Source: JAMA Netw Open. 2025 Feb 17;8(2):e2460283. doi: 10.1001/jamanetworkopen.2024.60283 (PMC11833519; doi:10.1001/jamanetworkopen.2024.60283)
Supplement: Supplement 2. — Data Sharing Statement [file jamanetwopen-e2460283-s002.pdf]

Data Sharing Statement

Zouiouich. Calcium Intake and Risk of Colorectal Cancer in the NIH-AARP Diet and Health Study. *JAMA Netw Open*. Published February 17, 2025.
doi:10.1001/jamanetworkopen.2024.60283

Data
Data available: Yes
Data types: Deidentified participant data, Data dictionary
How to access data: Data requests should be directed to the NIH-AARP Diet and Health Study (https://dietandhealth.cancer.gov/). Study resources, including data dictionaries and information on questionnaires, are available at the study websites cited above.
When available: With publication

Supporting Documents
Document types: Statistical/analytic code
How to access documents: Requests for analytic code can be sent to erikka.loftfield@nih.gov.
When available: With publication

Additional Information
Who can access the data: Data will be made available to researchers whose proposed use of the data has been approved.
Types of analyses: Data will be made available for research purposes.
Mechanisms of data availability: Data will be made available after approval of a proposal and with a signed data access agreement.
